# Physiological variations in hypovirus-infected wild and model long-term laboratory strains of *Cryphonectria parasitica*

**DOI:** 10.3389/fmicb.2023.1192996

**Published:** 2023-06-22

**Authors:** Maja Popović, Lucija Nuskern, Karla Peranić, Rosemary Vuković, Zorana Katanić, Ljiljana Krstin, Mirna Ćurković-Perica, Deborah Marie Leigh, Igor Poljak, Marilena Idžojtić, Daniel Rigling, Marin Ježić

**Affiliations:** ^1^Department of Biology, Faculty of Science, University of Zagreb, Zagreb, Croatia; ^2^Department of Biology, Josip Juraj Strossmayer University of Osijek, Osijek, Croatia; ^3^Swiss Federal Research Institute WSL, Birmensdorf, Switzerland; ^4^Faculty of Forestry and Wood Technology, University of Zagreb, Zagreb, Croatia

**Keywords:** culture deterioration, fungal laccases, fungal metabolism, long term sub-culturing, oxidative stress, virus-host interactions

## Abstract

**Introduction:**

Forest ecosystems are highly threatened by the simultaneous effects of climate change and invasive pathogens. Chestnut blight, caused by the invasive phytopathogenic fungus *Cryphonectria parasitica*, has caused severe damage to European chestnut groves and catastrophic dieback of American chestnut in North America. Within Europe, the impacts of the fungus are widely mitigated through biological control that utilizes the RNA mycovirus: Cryphonectria hypovirus 1 (CHV1). Viral infections, similarly to abiotic factors, can cause oxidative stress in their hosts leading to physiological attrition through stimulating ROS (reactive oxygen species) and NOx production.

**Methods:**

To fully understand the interactions leading to the biocontrol of chestnut blight, it is vital to determine oxidative stress damage arising during CHV1 infection, especially considering that other abiotic factors, like long-term cultivation of model fungal strains, can also impact oxidative stress. Our study compared CHV1-infected *C. parasitica* isolates from two Croatian wild populations with CHV1-infected model strains (EP713, Euro7 and CR23) that have experienced long-term laboratory cultivation.

**Results and Discussion:**

We determined the level of oxidative stress in the samples by measuring stress enzymes’ activity and oxidative stress biomarkers. Furthermore, for the wild populations, we studied the activity of fungal laccases, expression of the laccase gene *lac1*, and a possible effect of CHV1 intra-host diversity on the observed biochemical responses. Relative to the wild isolates, the long-term model strains had lower enzymatic activities of superoxide dismutase (SOD) and glutathione S-transferase (GST), and higher content of malondialdehyde (MDA) and total non-protein thiols. This indicated generally higher oxidative stress, likely arising from their decades-long history of subculturing and freeze–thaw cycles. When comparing the two wild populations, differences between them in stress resilience and levels of oxidative stress were also observed, as evident from the different MDA content. The intra-host genetic diversity of the CHV1 had no discernible effect on the stress levels of the virus-infected fungal cultures. Our research indicated that an important determinant modulating both *lac1* expression and laccase enzyme activity is intrinsic to the fungus itself, possibly related to the vc type of the fungus, i.e., vegetative incompatibility genotype.

## 1. Introduction

The most important stress factors affecting contemporary forest ecosystems are not only climate change related, but also include pressure from invasive pathogenic species (Trumbore et al., 2015). Invasive phytopathogenic fungi have caused significant damage to several important tree species. The most well-known examples of such pathogens include *Ophiostoma novo-ulmi* (Brasier and Buck, 2001; Solla et al., 2008; Katanić, 2020), *Hymenoscyphus fraxineus* (Pautasso et al., 2013; Burokiene et al., 2015) and *Cryphonectria parasitica* (Anagnostakis, 1987; Rigling and Prospero, 2018). Each fungal pathogen has been responsible for the serious dieback of trees in Europe and North America, jeopardizing forest ecosystems. This is particularly notable for chestnut blight, caused by *C. parasitica*, which led to the functional extinction of the North American chestnut tree and severe damage to European chestnut groves.

Within Europe, chestnut blight is now mediated by natural biological control that utilises the RNA mycovirus Cryphonectria hypovirus 1 (CHV1) (MacDonald and Fulbright, 1991; Heiniger and Rigling, 1994). This biocontrol is highly successful, as it allows the trees to survive the fungal attack, has no secondary environmental impacts, and is self-sustaining due to chronic infections of the fungus and natural spread of the virus.

CHV1 infection undoubtedly exerts biotic stress on the host fungus which has been confirmed in model long-term laboratory strains (Allen et al., 2003; Kim et al., 2012; Nuskern et al., 2017), but understanding of the virus-induced stress in naturally occurring (wild) hypovirulent strains is scarce. Furthermore, most studies were performed using one or a limited number of laboratory model strains. Long-term laboratory culturing conditions have been shown in many systems to introduce artefacts that are not present in the natural environment, e.g., *in vitro* cell culture (Olsavsky et al., 2007; Kim et al., 2012; Li et al., 2014).

Reactive oxygen species (ROS) are generated either in low concentrations to engage in many important cell processes and signaling pathways, or pathologically where high ROS concentrations can cause significant cellular damage (da Silva Caetano et al., 2019; Almeida et al., 2020). The increase in ROS concentrations inside the cell and/or the reduction of the cell’s antioxidant capacity leads to the disruption of the redox control, resulting in oxidative stress (da Silva Caetano et al., 2019). Thus, controlling ROS levels is essential to maintain cellular homeostasis. This is ensured by the cellular antioxidant system which is comprised of enzymes such as superoxide dismutase (SOD), catalase (CAT), glutathione peroxidase (GPx), and non-enzymatic antioxidants including vitamin C, vitamin E, carotenoids, glutathione (GSH) and flavonoids (da Silva Caetano et al., 2019). Like plants and animals, fungi produce several antioxidative enzymes like CAT, SOD, GPx and glutathione reductase to counteract oxidative stress caused by ROS (Jamieson, 1998; Azevedo et al., 2007; Breitenbach et al., 2015), but alteration of their activity during virus infection is still understudied (Nuskern et al., 2017). As obligate intracellular parasites, viruses can interfere with the cell’s ROS homeostasis and modulate the host’s antioxidant defences, contributing to overall pathogenesis (Reshi et al., 2014). Viral infections usually stimulate ROS production in cells, which leads to increased levels of oxidative stress biomarkers and a change in the activities of antioxidant enzymes (Reshi et al., 2014; Camejo et al., 2016; da Silva Caetano et al., 2019; Almeida et al., 2020). Oxygen radicals and NO_x_, generated in excess during oxidative stress have been implicated in the generation of point mutations in a viral RNA genome and contribute to the high diversity of viral intra-host populations (Akaike et al., 2000; Akaike, 2001). However, research on viral intra-host genetic diversity is relatively scarce as often only human and plant-crop pathogens are explored (Lauring and Andino, 2010; Simmons et al., 2011; Dunham et al., 2014; Lauring, 2020; Feder et al., 2021; Leigh et al., 2021).

The pathosystem *C. parasitica* – CHV1 has long been used as a model for studying mycovirus biology, hypovirulence, virus-host interactions, fungal physiology, and biological disease control e.g. (Chen and Nuss, 1999; Peever et al., 2000; Kim et al., 2002; Hillman and Suzuki, 2004; Dawe and Nuss, 2013; Muñoz-Adalia et al., 2016). The early work on CHV1 cDNA cloning, sequence analysis and genomic organization was conducted only on the virus strain CHV1-EP713 (Shapira et al., 1991). Now considered “prototypic strain” it has been used in many studies of hypovirus effects on the host e.g. (Choi et al., 1992; Allen et al., 2003; Dawe et al., 2003; Wang et al., 2016). In addition to CHV1-EP713, CHV1-Euro7 has been studied in detail and established as a comparative prototypic strain (Chen and Nuss, 1999; Gobbin et al., 2003; Allen and Nuss, 2004).

Notably, these two CHV1 prototypic strains were isolated as early as the 1970s (Allemann et al., 1999; Chen and Nuss, 1999), and have been subsequently subculturing, stored freeze-dried or as glycerol stocks, and shipped to be shared between laboratories. These processes are unlikely to be benign to both, the virus and fungal host. In particular, the freeze/thaw cycles are likely a source of stress for the fungal host. In other species of fungi, long-term subculturing has been shown to lead to morphological heterogeneity, culture degeneration, physiological changes and reduced viability of the frozen stock (Ryan et al., 2002; Li et al., 2014; Jukic et al., 2017; Cabrera et al., 2020). Consequently, some conclusions about fungal physiology derived from the experiments on long-term *C. parasitica* model strains and prototypic hypovirus strains may not accurately reflect natural populations. For example, fungal enzymes such as laccases, considered a hallmark of pathogenic potential of *C. parasitica,* are important in many other biological processes including development of fungal structures, pigment synthesis, lignin decomposition and fungal/plant interactions (Baldrian, 2006) but previous studies have shown inconsistent results regarding changes in the laccase activity across different strains and experimental settings (Rigling et al., 1989; Rigling and Van Alfen, 1991; Allen et al., 2003; Gong et al., 2017; Nuskern et al., 2021). Most recently the intra-host populations of CHV1 from naturally infected Swiss and Croatian *C. parasitica* isolates were characterised (Leigh et al., 2021). These isolates offer an interesting opportunity to compare physiological differences between ‘wild’ cultures and ‘laboratory’ model strains of the *C. parasitica* – CHV1 system, in order to demonstrate the impacts of long-term manipulation.

The aims of this study were to (i) compare the oxidative stress parameters between naturally CHV1-infected *C. parasitica* isolates and CHV1-infected long-term laboratory model strains; (ii) determine the level of oxidative stress in two wild *C. parasitica* populations naturally infected with CHV1, by measuring stress enzymes activity and oxidative stress biomarkers; (iii) compare the enzyme activity of fungal laccases and the expression of the laccase gene *lac1* in naturally infected populations; (iv) determine the possible effect of CHV1 intra-host diversity on the observed biochemical response of naturally infected fungus.

## 2. Materials and methods

### 2.1. Wild populations

#### 2.1.1. Sample collection

To obtain samples naturally infected with CHV1, two wild *Cryphonectria parasitica* populations were sampled in July 2019 from two chestnut growing areas in Croatia, Ozalj (45.59°N, 15.46°E, elevation approx. 220–320 m) and Kast (45.70°N, 15.36°E, elevation approx. 540–640 m). The fungal populations’ diversity and CHV1 prevalence were previously determined and published (Ježić et al., 2021; Leigh et al., 2021). Fifty chestnut trees infected with *C. parasitica*, which causes bark wounds called cankers, were randomly chosen in each population and only one canker per tree was sampled. A bark plug was collected from each canker with a 2 mm bone marrow biopsy needle, which was sterilized after each sampling by dipping in 96% ethanol and flaming [see (Leigh et al., 2021) for full sampling methods].

#### 2.1.2. Laboratory culturing

Bark plugs were surface sterilized by dipping them in 70% ethanol for several seconds, then drying on sterile filter paper, after which they were placed in φ 90 mm Petri plates containing 20 mL of potato dextrose agar (PDA) (Difco, BD, Franklin Lakes, NJ), followed by incubation in a growth chamber at 24°C and 70% humidity in the dark. After several days, most of the freshly grown mycelium expanding from bark samples was transferred to four new Petri plates with fresh PDA overlaid with cellophane. This was done to preserve most of the CHV1 population variability within each sample. After 10 days of growth, mycelia from all four Petri plates were stripped from the cellophane and the samples were combined into a single composite sample, transferred to previously weighted 2 mL tubes and lyophilized. All composite samples were used for RNA and DNA extraction (hypovirulence assessment and *vic* genotyping).

#### 2.1.3. CHV1 detection

The virus was detected by RT-PCR using dsRNA as template. For dsRNA extraction, the Double-RNA Viral dsRNA extraction mini kit (iNtRON Biotechnology Inc., Seongnam, South Korea) was used. First-strand cDNA synthesis was conducted using the GoScript™ Reverse Transcription System (Promega, Madison, WI, USA) or High-Capacity cDNA Reverse Transcription Kit with RNase Inhibitor (Applied Biosystems by Thermo Fisher Scientific Waltham, MA, USA), with 10 μL of isolated dsRNA. The PCR reaction was performed as described by Allemann et al. (1999) and the obtained amplicons separated on 1% (wt/vol) agarose gel in 0.5× TBE buffer at 5 V/cm and visualized with GelStar DNA stain (Lonza, Basel, Switzerland).

#### 2.1.4. Vegetative compatibility genotyping of *Cryphonectria parasitica*

Vegetative compatibility (vc) genotyping was done first by pairing all samples with tester strains of the most abundant vc types in Croatia – EU-1 and EU-2, as described in (Krstin et al., 2008). For all isolates which could not be unequivocally assigned to either of the aforementioned EU types, molecular *vic* genotyping was performed. DNA extractions were done with OmniPrep™ for Fungi extraction kit (G-Biosciences, Saint Louis, MO, USA), PCR reaction were performed as described in Mlinarec et al. (2018a) and the obtained amplicons separated on 1.5% (wt/vol) agarose gel in 0.5× TBE buffer at 5 V/cm and visualized with GelStar DNA stain (Lonza, Basel, Switzerland).

From each population, 10 CHV1-infected isolates were randomly chosen for subsequent genetic and biochemical analyses. Intra-host diversity of CHV1 was successfully determined by PacBio long-read HiFi sequencing for 10 isolates from the population Ozalj and nine from Kast, as published in Leigh et al. (2021). Those previously characterised isolates were further used in this study for biochemical analyses.

### 2.2. Model strains

Besides the *C. parasitica* samples from the wild populations, we used three well-characterized, long-term subculturing, CHV1-infected *C. parasitica* model strains (hereafter referred to as “model strains”) regenerated from stock cultures kept at −80°C in 22% glycerol. Those were the model strains harboring the prototypic CHV1 strains EP713 and Euro7, as well as isolate CR23 – a Croatian CHV1-infected field isolate of *C. parasitica* collected in the early 2000s, whose CHV1 genome has been fully sequenced (Krstin et al., 2008; Mlinarec et al., 2018b). Growth conditions followed those used for the field isolates from the wild populations (described in 2.1.2): small agar blocks of model strains tissue stocks stored at −80°C were placed in φ 60 mm Petri plates containing *ca.* 10 mL PDA, followed by incubation in a growth chamber at 24°C and 70% humidity in the dark. After several days of growth, model strains colonies were subcultured to four new φ 90 mm Petri plates containing 20 mL PDA overlaid with cellophane. After 10 days of growth, mycelia were stripped from the cellophane, combined into a single composite sample, transferred to previously weighted 2 mL tubes and lyophilized for further analysis.

### 2.3. Oxidative stress parameters of *Cryphonectria parasitica*

#### 2.3.1. Preparation of protein extracts

Lyophilized samples were ground to a fine powder with φ 5 mm steel ball in TissueLyser II (Qiagen, Venlo, Netherlands) for 2 min at 30 Hz. From each ground sample, three replicates were prepared and weighed separately. To prepare the protein extracts for measurement of the level of lipid peroxidation and the activities of GST, SOD and LAC, 1750 μL of cold extraction buffer (100 mM potassium phosphate buffer, with 0.1 mM EDTA, pH 7.0) was added to the ground lyophilized tissue, mixed with a micro pestle and vortexed briefly. The homogenate was centrifuged at 20000× *g* for 20 min at 4°C. Supernatant was collected and protein concentration was determined according to Bradford (1976) using bovine serum albumin (Protein Standard, 2 mg/vial BSA, Sigma-Aldrich® Burlington, MA, United States) as a standard. The samples were aliquoted and stored at −20°C. All spectrophotometric measurements were done with a Multiscan Sky microplate spectrophotometer (Thermo Scientific™ ver 5.0) in 96-well plates (Nunc MaxiSorp™ flat-bottom, Thermo Scientific™).

#### 2.3.2. Stress enzymes and laccase activity measurements

The activity of glutathione S-transferase (GST) was assayed spectrophotometrically as described in Bocchetti and Regoli (2006). The increase in absorbance at 340 nm was measured 10 times every 10 s, using 1-chloro-2,4-dinitrobenzene (CDNB) and reduced glutathione (GSH) as substrates. The reaction mixture was 100 mM potassium phosphate buffer (pH 6.4) with 1 mM CDNB. The reaction was done in plates by pipetting 277.5 μL of the reaction mixture, 7.5 μL of 100 mM GSH (final concentration was 2.5 mM) and 15 μL of the sample. GST activity was expressed in nanomoles of generated GS-DNB per minute per milligram of dry weight (DW) (*ε* = 9.6 mM^−1^ cm^−1^).

The activity of superoxide dismutase (SOD) was determined based on the inhibition of the photochemical reduction of nitroblue tetrazolium (NBT, Sigma-Aldrich) at 560 nm as described by Beauchamp and Fridovich (1971). The reaction mixture was 50 mM potassium phosphate buffer (pH 7.8) with 75 μM NBT and 0.1 mM EDTA. The reaction was done by pipetting 247.5 μL of the reaction mixture, 22.5 μL of 10.8 mM xanthine (final concentration was 0.81 mM), 10 μL of extraction buffer, 15 μL of 0.05 U/mL XOD (final concentration was 2.5 mU/mL) and 5 μL of the sample. The uninhibited reaction (max) contained 15 μL of extraction buffer instead of the sample. After mixing all reactants the plate was shaken for 5 min at low speed, and the absorbance was measured after 10 min. The inhibition of the reaction by SOD (%) was calculated as:


$$\%inh=\frac{\left(Amax-Ablank\right)-\left(Asample-Ablank\right)}{\left(Amax-Ablank\right)}*100$$

SOD activity was calculated from the calibration curve, obtained from % inhibition data plotted against log-SOD concentration, using bovine recombinant SOD (expressed in *Escherichia coli*, Sigma-Aldrich) as standard. One unit of SOD activity was defined as the amount of enzyme which caused 50% inhibition of NBT reduction. Results were expressed as enzyme units per milligram of dry weight (DW).

Laccase activity (LAC) was measured as an increase in absorbance at 468 nm due to 2,4-dimethoxyphenol DMOP oxidation to 3,3′,5,5′-tetramethoxydiphenylquinone (DMOPox, ε = 14.8 mM^−1^ cm^−1^) combining the methods described by Rigling et al. (1989) and Palmieri et al. (1997). The final reaction mixture consisted of 240 μL of citrate–phosphate buffer (0.1 M Na_2_HPO_4_ and 0.05 M citric acid, pH 3.4), 15 μL of 50 mM DMOP (prepared in the same citrate–phosphate buffer; final concentration 2.5 mM) and 45 μL of protein extract. The activity was expressed in nmol of the generated DMOPox per minute per mg of dry weight (DW).

#### 2.3.3. Measurement of oxidative stress biomarkers

The content of total non-protein thiols (THIOLS) was determined using Ellman’s method (Ellman, 1959). Lyophilized ground tissue was homogenized with 1 mL 6.67% sulfosalicylic acid, incubated for 30 min on ice and centrifuged for 10 min at 13000× *g* at 4°C to precipitate proteins. Afterwards, supernatants (600 μL) were mixed with 150 μL of 5 mM 5,5′-dithiobis (2-nitrobenzoic acid), (DTNB; final concentration 1 mM), incubated for 15 min at room temperature, followed by measurement of absorbance at 412 nm. The non-protein thiol content was determined by the use of an extinction coefficient *ε* = 14.53 mM^−1^ cm^−1^ and expressed in nmol of the generated DTNB per mg of dry weight (DW).

Lipid peroxidation was determined by estimating the malondialdehyde (MDA) content using the thiobarbituric acid (TBA) method described by Heath and Packer (1968). First, to precipitate proteins, 500 μL of protein extracts were mixed with 100% (w/v) trichloroacetic acid (TCA) to a final concentration of 10% TCA, incubated for 30 min on ice and then centrifuged for 10 min at 13000× *g* at 4°C. 500 μL of supernatant was mixed with the same volume of MDA reagent, consisting of 0.5% (w/v) TBA in 10% (w/v) TCA resulting in a final concentration of TBA of 0.25% (w/v). The mixture was heated at 95°C for 30 min, after which the reaction was stopped in an ice bath. The cooled reaction mixture was centrifuged at 10000× *g* for 10 min at 4°C to remove any leftover precipitate. MDA content in nmol per mg of dry weight (DW) was calculated from the absorbance at 532 nm, corrected for unspecific turbidity by subtracting the absorbance at 600 nm, by using an extinction coefficient *ε* = 155 mM^−1^ cm^−1^.

### 2.4. Expression of the laccase gene *lac1*

#### 2.4.1. RNA isolation and cDNA synthesis

Total RNA was extracted from lyophilized mycelia using the NucleoZOL reagent (Macherey-Nagel, Düren, Germany), following the manufacturer’s instructions. RNA concentration and purity were determined using a NanoPhotometer NP80 (Implant, München, Germany), while RNA integrity was verified by agarose gel electrophoresis and SYBR safe staining (Invitrogen, Carlsbad, CA, USA). The average RNA yield was around 1,500 ng/μL, while A260/280 ratio was approximately 1.9–2.0. Before cDNA synthesis, residual DNA in the obtained RNA solution was removed by RQ1 RNase-Free DNase (Promega, Madison, WI, USA). First-strand cDNA was synthesized from 3 μg of total RNA by the GoTaq® 2-Step RT-qPCR System (Promega, Madison, WI, USA) according to the manufacturer’s recommendation using a random hexamer mixture provided in the kit.

#### 2.4.2. qPCR of *lac1*

Quantitative PCR (qPCR), using dye-based detection, was performed to analyse transcript levels of the *lac1* gene. Gene encoding beta-tubulin was used as a reference gene for normalization. The oligonucleotide primers for laccase (*lac1*-FW: ATCAATCCGGCTAACACGAC; REV: TGTCATAGAATGGCCCACAA) and beta-tubulin (*tubulin*-FW: AGTGGATTCCCAACAACGTC; REV: CTTGAAGAGCTCCTGGATGG) were designed based on sequences in the GeneBank database (accession no. M73257 for *lac1*, accession no. AH014582 for *tubulin*) using Primer3 software. qPCR analysis was performed on StepOnePlus™ Real-Time PCR System with StepOnePlus™ Software v2.3 (Applied Biosystems, Waltham, MA, USA) and by using GoTaq® 2-Step RT-qPCR System (Promega, Madison, WI, USA), according to the manufacturer’s recommendation. For qPCR analysis, cDNA was diluted two-fold and measured in three technical replicates. Standard curves based on five points, corresponding to a twofold dilution series from selected cDNA served as standard and were used to compute the PCR efficiency of each primer pair. The average PCR efficiencies were 100% for the *tubulin* gene and 97.5% for the *lac1* gene, while *R*^2^ values calculated for standard curves were 0.98 and 0.99 for the *tubulin* and *lac1* genes, respectively. The specificity of the qPCR reaction was confirmed by melting curve analysis, and the relative expression of the *lac1* gene was quantified by the comparative 2^−ΔΔCt^ method (Pfaffl, 2001).

### 2.5. CHV1 intra-host population variability

For the same wild population samples used in this study, the genetic diversity of intra-host CHV1 populations was determined previously by Leigh et al. (2021). Briefly, Nei’s H was calculated to characterize CHV1 intra-host haplotype diversity (Nei and Tajima, 1980) and intra-host mutational diversity was measured using nucleotide diversity, i.e., π, estimated in SNPGenie (Nelson et al., 2015). In this research, we used those previously obtained viral variant diversity data to compare them with oxidative stress parameters of the same host isolates.

### 2.6. Data analysis of oxidative stress parameters

Statistical analyses were done with Statistica 13 (StatSoft Inc., Tulsa, OK, USA). Data of biochemical analysis are presented as mean ± standard error. Each data point on an individual level is the average of three replicates (*n* = 3). The normality of the data was tested by Kolmogorov–Smirnov and Lilliefors test of normality. The estimation of relationships between analyzed variables was done with Pearson’s correlation coefficient, at *p* < 0.05. To determine the differences between the population of wild isolates and model strains for measured physiological stress parameters *t*-test was used. Factorial ANOVA was used to determine which factors affect stress-related parameters between isolates from wild populations, followed by *post hoc* Tukey honest significant difference (HSD) test. For this, we analyzed the oxidative stress parameters by grouping the samples according to (1) their population of origin (Ozalj or Kast) or (2) vc type: EU-1 or EU-2 which are the two most abundant vc types in Croatia, or “EU-other” (comprised of all other samples represented by a single isolate of a particular vc type: e.g. EU3, EU5, EU13, EU15).

## 3. Results

### 3.1. Model strains vs. wild isolates

**Figure 1:**
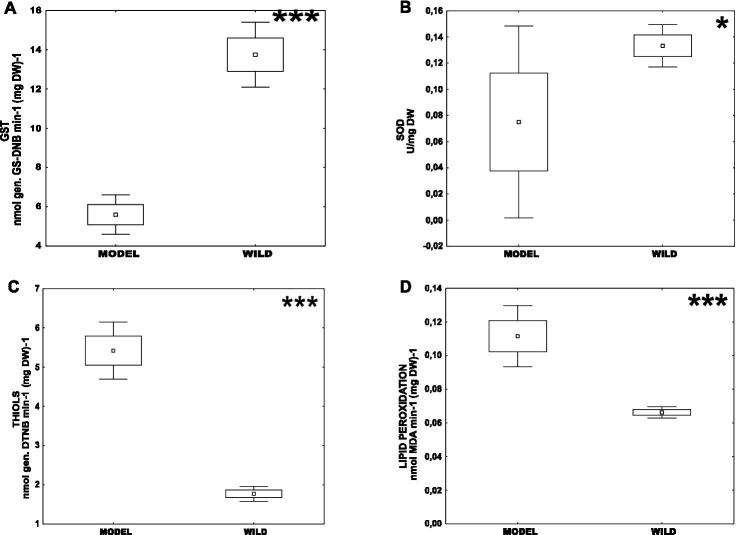
Difference of oxidative stress parameters between CHV1-infected long-term laboratory model strains (MODEL) – Euro7, EP713, CR23 and wild populations naturally infected with CHV1 – Kast and Ozalj (WILD) for: **(A)** activity of GST, **(B)** activity of SOD, **(C)** content of non-proteins thiols and **(D)** level of lipid peroxidation. Data are represented as mean, mean ± SE (box), mean ± 1.96 SE (Whiskers). Statistically significant differences are denoted by asterisks as follows: *p < 0.05; **p < 0.001; ***p < 0.0001.

First, we analyzed the difference in the oxidative stress parameters between CHV1-infected long-term laboratory model strains – Euro7, EP713, CR23 (MODEL) and population isolates naturally infected with CHV1 – Kast and Ozalj (WILD). Those two groups differed significantly in all measured parameters, i.e., the activity of GST and SOD, content of non-protein thiols, and level of lipid peroxidation (Figure 1.). The enzymatic activities were significantly higher in wild populations [mean(GST) = 13.77 ± 0.79 nmol (GS-DNB) min^−1^ (mg DW)^−1^; mean(SOD) = 0.13 ± 0.01 U (mg DW)^−1^] than in model strains [mean(GST) = 5.80 ± 1.38 nmol (GS-DNB) min^−1^ (mg DW)^−1^; mean(SOD) = 0.07 ± 0.02 U (mg DW)^−1^]. On the other hand, the content of non-protein thiols and the level of lipid peroxidation were significantly higher in model strains [mean(THIOLS) = 5.41 ± 0.25 nmol (DTNB) (mg DW)^−1^; mean(MDA) = 0.11 ± 0.01 nmol (mg DW)^−1^] than in wild populations [mean(THIOLS) = 1.75 ± 0.14 nmol (DTNB) (mg DW)^−1^; mean(MDA) = 0.06 ± 0.00 nmol (mg DW)^−1^]. For model strains, there was a significant positive correlation between MDA content and SOD activity (0.695), the content of total non-protein thiols and SOD activity (0.622) and the content of MDA and total non-protein thiols (0.576), all at *p* < 0.05.

### 3.2. Wild *Cryphonectria parasitica* populations

To test the effect of the CHV1 intra-host diversity on the host physiology we compared oxidative stress parameters of fungal isolates with parameters of viral genetic diversity (Nei’s H and π) for the CHV1 populations from the same isolates. We found no statistically significant correlation between Nei’s H and any of the tested parameters, while π slightly negatively correlated with SOD (−0.3619) and positively with lipid peroxidation (0.3823) at *p* < 0.05 (Supplementary Figures 1, 2). However, individual samples like KS 06_3, which had the highest genetic diversity of CHV1 also had the highest values for the activity of GST, the content of thiols and the level of lipid peroxidation. Otherwise, isolate KS 11_2 which had the lowest CHV1 diversity also had among the lowest values for analyzed oxidative stress parameters.

**Figure 2:**
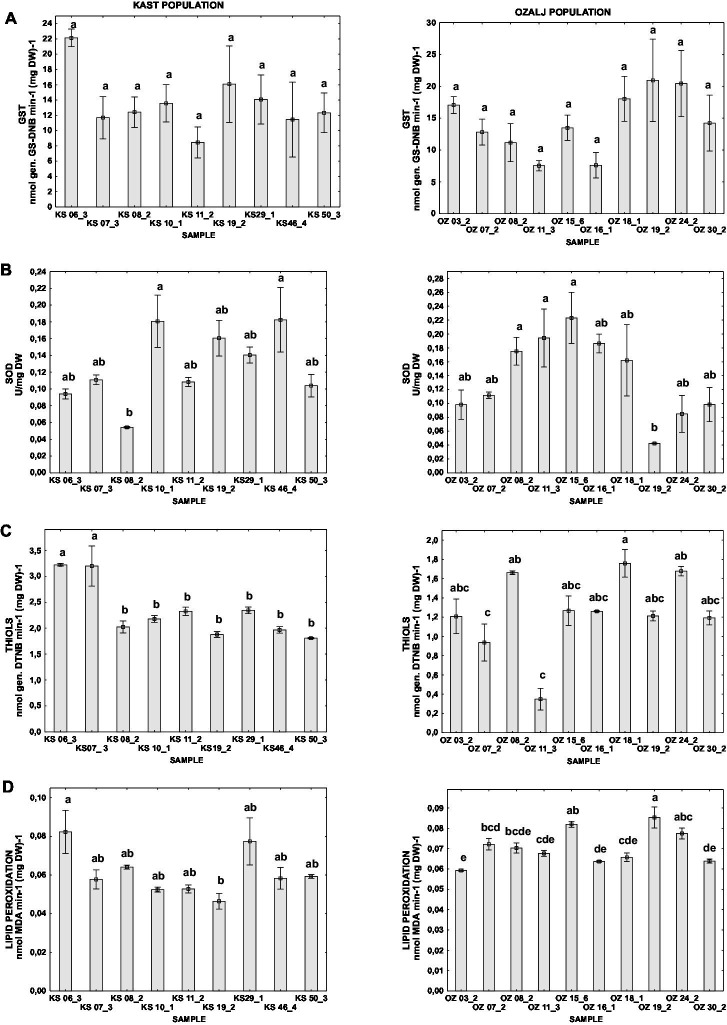
Difference of oxidative stress parameters for individual isolates of Kast (KS) and Ozalj (OZ) populations naturally infected with CHV1: **(A)** activity of GST, **(B)** activity of SOD, **(C)** content of non-proteins thiols, and **(D)** level of lipid peroxidation. Data are presented as mean ± SE. Letters denote the statistical difference between individual isolates for each population (Tukey HSD), respectively, at *p* < 0.05.

While the intra-population differences in oxidative stress parameters between samples were noticeable, ANOVA followed by *post hoc* Tukey HSD test indicated statistically significant differences between some samples in all parameters except for GST (Figure 2A), which might be explained by the large variability of the measured parameter in both populations.

**Figure 3:**
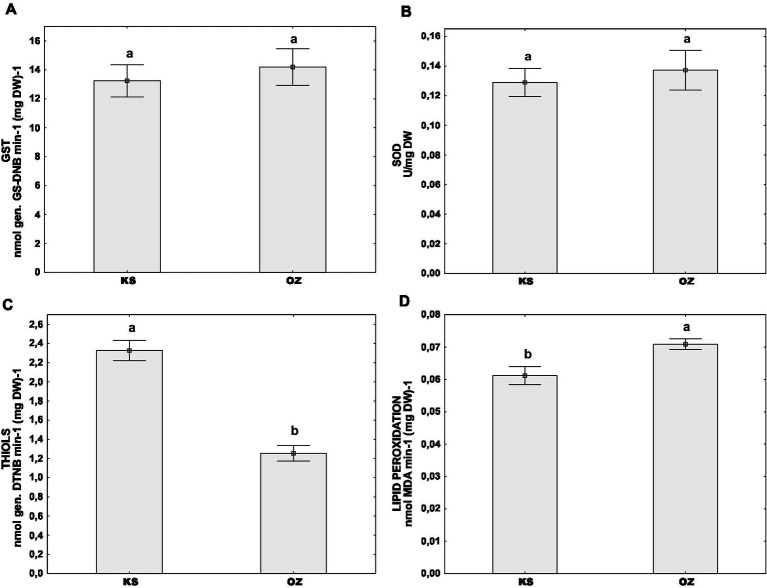
Difference of oxidative stress parameters between two wild populations naturally infected with CHV1 – Kast (KS) and Ozalj (OZ) for: **(A)** activity of GST, **(B)** activity of SOD, **(C)** content of non-proteins thiols and **(D)** level of lipid peroxidation. Data are represented as mean ± SE. Letters denote the statistical difference between individual isolates for each population (Tukey HSD), respectively, at *p* < 0.05.

To test the possible effect of each isolates’ vc type or population of origin on the stress parameters factorial ANOVA was used. The activity of GST or SOD was not influenced by either of the two aforementioned factors (Table 1; Figures 3A,B). Surprisingly, total thiol content was affected by both studied factors (i.e., the population of origin and the vc type of the isolate) and lipid peroxidation level by isolates’ vc type. The population Kast had a higher content of thiols [mean(THIOLS) = 2.33 ± 0.10 nmol (DTNB) (mg DW)^−1^] and lower level of lipid peroxidation [mean(MDA) = 0.06 ± 0.00 nmol (mg DW)^−1^] in comparison to the population Ozalj [mean(THIOLS) = 1.25 ± 0.10 nmol (DTNB) (mg DW)^−1^; mean(MDA) = 0.07 ± 0.00 nmol (mg DW)^−1^] (Table 1; Figures 3C,D).

**Table 1:** Factorial ANOVA – Univariate test of significance for oxidative stress parameters between different groups of isolates based on their population of origin (Population), vc type (vc type), or the combination of both (Population * vc type).

Effect	GST				SOD			
	df^a^	MS^b^	*F*	*p*	df^a^	MS^b^	*F*	*p*
Population	1	13.423	0.3435	0.560495	1	0.003125	0.8165	0.370704
vc type	2	61.034	1.562	0.219981	2	0.00111	0.2901	0.749497
Population * vc type	2	86.22	2.2065	0.120902	2	0.004319	1.1283	0.332003
Effect	Total non-protein thiols				Lipid peroxidation			
	df^a^	MS^b^	*F*	*p*	df^a^	MS^b^	*F*	*p*
Population	1	14.087	88.962	0***	1	0.000188	1.819	0.183541
vc type	2	0.4407	2.7834	0.071426	2	0.001244	12.028	0.000054***
Population * vc type	2	2.4655	15.57	0.000006*	2	0.000359	3.475	0.038636*

Statistically significant differences are denoted by asterisks as follows: *p < 0.05; **p  < 0.001; ***p<0.0001. ^a^degrees of freedom. ^b^mean square value.

**Figure 4:**
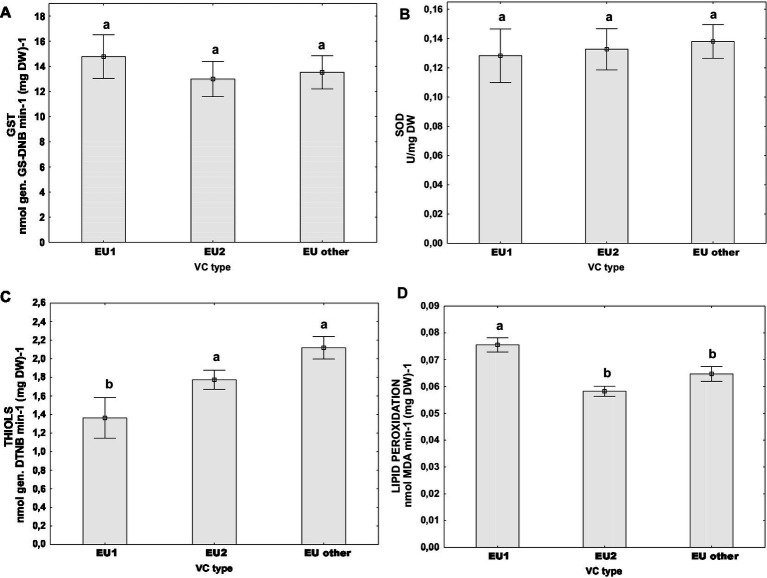
Difference of oxidative stress parameters for populations naturally infected with CHV1 grouped by vc types (EU-1, EU-2, “EU-other”): **(A)** activity of GST, **(B)** activity of SOD, **(C)** content of non-proteins thiols, and **(D)** level of lipid peroxidation. Data are represented as mean ± SE. Letters denote the statistical difference between vc type groups (Tukey HSD), at *p* < 0.05.

Furthermore, we analyzed the oxidative stress parameters by grouping the samples according to their vc type: EU-1 or EU-2 which are the two most abundant vc types in Croatia, or “EU-other” (comprised of all other vc types, eg. EU-3, EU-5, EU-13 and EU-15). *Post hoc* Tukey HSD test did not indicate that the activities of measured stress-related enzymes GST and SOD were significantly different between the aforementioned vc type groups (Figures 4A,B). However, we noticed significant differences in the content of non-protein thiols (Figure 4C) and the level of lipid peroxidation (Figure 4D) between vc types. Isolates of the vc type EU-1 had the lowest content of thiols [mean(THIOLS) = 1.36 ± 0.16 nmol (DTNB) (mg DW)^−1^), but the highest level of lipid peroxidation (mean(MDA) = 0.08 ± 0.00 nmol (mg DW)^−1^], significantly different from both parameters in two other groups: EU-2 and “EU-other.” On the other hand, EU-2 and “EU-other” were similar in both the content of total thiols – for EU-2: [mean(THIOLS) = 1.77 ± 0.16 nmol (DTNB) (mg DW)^−1^], for “EU-other”: [mean(THIOLS) = 2.12 ± 0.15 nmol (DTNB) (mg DW)^−1^], and the level of lipid peroxidation – for EU-2: [mean(MDA) = 0.05 ± 0.00 nmol (mg DW)^−1^] and EU-other: [mean(MDA) = 0.06 ± 0.00 nmol (mg DW)^−1^].

### 3.3. Laccase activity and *lac1* gene expression

To assess the physiology of naturally CHV1-infected *C. parasitica*, we measured the activity of fungal laccases. In the population Kast, the mean value of laccase activity was 0.79 ± 0.05 nmol (DMOP) min^−1^ (mg DW)^−1^ (0.40–1.29 nmol (DMOP) min^−1^ (mg DW)^−1^), and in Ozalj the mean value was 1.06 ± 0.12 nmol (DMOP) min^−1^ (mg DW)^−1^ (0.29–2.73 nmol (DMOP) min^−1^ (mg DW)^−1^). The *t*-test showed no difference between populations. Similarly, the *t*-test did not indicate a significant difference in the relative expression of the *lac1* between the two populations which ranged from 0.245 to 1.850 in Kast and from 0.027 to 2.425 in Ozalj.

**Figure 5:**
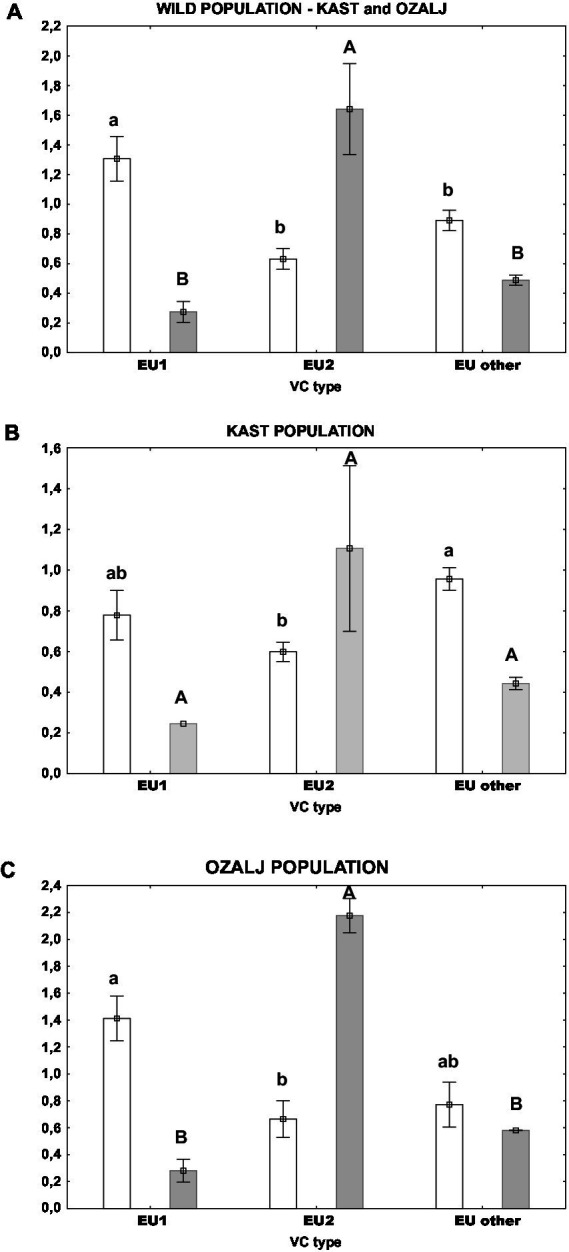
Laccase activity (white columns) and *lac1* relative expression (grey columns) in isolates of different vc types: **(A)** both populations, **(B)** Kast population, **(C)** Ozalj population. Standard errors are indicated with error bars. Lower case letters denote statistical significance among samples for laccase activity; upper case letters denote statistical significance among samples for *lac1* relative expression; Tukey HSD, all at *p* < 0.05.

Furthermore, we measured the activity of fungal laccases and the relative expression level of *lac1* encoding extracellular laccase1 in naturally infected populations. Univariate tests of significance singled out the vc type of the fungus as a factor that has a statistically significant effect on both parameters. Thus, we further analyzed the laccase parameters by grouping the samples according to their vc type: EU-1, EU-2 and “EU-other,” as previously discussed. As shown in Figure 5. the relation between laccase activity and *lac1* relative expression is opposite and inversely proportional between EU-1 and EU-2 vc types. If we analyse samples from both wild populations (Figure 5A), we see that the laccase activities were significantly higher in samples of EU-1 than of EU-2 vc type, while the *lac1* relative gene expression was significantly lower for EU-1 vc type in comparison to EU-2. The trend is the same if we separate respective populations (Figures 5B,C), but the significance is lost for the Kast population (Figure 5B) due to the large data scatter.

## 4. Discussion

The present study broadens our knowledge of the physiology and stress response of a mycovirus-infected phytopathogenic fungus recently obtained from its natural habitat, versus physiological specificities of virus-infected long-term model strains. We have demonstrated clear differences in the physiology between wild populations and model strains; notably model strains had lower enzymatic activities of SOD and GST and higher content of MDA (i.e., level of lipid oxidation) and total non-protein thiols. These results indicate that the model strains are generally exposed to higher oxidative stress than the wild *C. parasitica* strains naturally infected with CHV1, i.e., high levels of lipid peroxidation induced by ROS (Birben et al., 2012) and the reduced SOD and GST activities suggest the depletion in cellular antioxidative enzymatic systems (Almeida et al., 2020). A similar correlation between the increase in MDA content and a decrease in SOD activity has been reported for Zika virus-infected cells, both *in vitro* and *in vivo* (Almeida et al., 2020). Additionally, a decrease in GST activity was observed in pathogenic yeast *Trichosporon asahii* exposed to other types of stressors like heavy metals (Ilyas et al., 2014). We have also observed the increase in the total non-protein thiols content, i.e., a gross estimate of GSH abundance (Noctor et al., 2011), whose secondary induction can compensate for the decrease in SOD activity under oxidative stress (Aquilano et al., 2014), further corroborating our observation that the model strains are under higher stress levels compared to wild populations.

Although virus infections undoubtedly lead to the increase of cellular ROS content (Reshi et al., 2014) and the CHV1 stress induction in *C. parasitica* has been reported previously (Nuskern et al., 2017), the high levels of oxidative stress observed in model strains cannot be assigned to CHV1 infection alone, since wild *C. parasitica* populations studied here were infected as well. A possible explanation of the observed phenomenon might be the ageing and deterioration of the model strains, since *C. parasitica* infected with prototypic CHV1 strains have been subculturing and exposed to freeze–thaw cycles for decades. In a study on the ageing of stationary cultures of the yeast *Saccharomyces cerevisiae,* the decrease in SOD activity accompanied by the oxidative damage of cellular proteins was observed, suggesting an increased exposure of macromolecules to ROS during ageing (Jakubowski et al., 2000). Similarly, in a filamentous fungus *Aspergillus nidulans* there was a correlation between fungal culture deterioration and cellular oxidative stress as well as a down-regulation of a SOD homolog in mitochondria of degenerated culture sectors (Li et al., 2014). Thus, thorough consideration of the physiological specificities of model laboratory strains is needed, especially when extrapolating the results to wild populations. Similar discrepancies have been observed previously when comparing the efficacy of horizontal CHV1 transmissions between different laboratory settings and natural conditions on chestnut trees (Deng et al., 2007; Brusini and Robin, 2013; Zhang and Nuss, 2016; Stauder et al., 2019).

While we have observed differences between oxidative stress levels of populations Ozalj and Kast, our work did not indicate that the CHV1 intra-host diversity had any effect on it (Leigh et al., 2021), but rather that these differences might be caused by some other factor, such as the accumulation of viral genome in the cytoplasm of the fungus, the genetic composition of the fungus or even the host tree (Dutech et al., 2010; Heller and Tudzynski, 2011; Ježić et al., 2014; Montibus et al., 2015; Ortiz-Urquiza and Keyhani, 2015; Poljak et al., 2017; Ježić et al., 2021).

It seems that the aforementioned differences in stress levels between *C. parasitica* strains from Ozalj and Kast can be associated with the genetic background of these populations. The higher stress resilience, or lower overall levels of oxidative stress in strains from Kast is evident by the lower level of lipid peroxidation observed in the strains from that population. Furthermore, the relatively higher content of non-protein thiols in this population might have a protective role against oxidative stress damage (Bai et al., 2003). Thus, when we analyzed isolates grouped by their vc type (EU-1, EU-2, EU-other), vc types EU-2 and “EU-other” had lower levels of lipid peroxidation and relatively higher content of non-protein thiols in comparison to EU-1. However, the prevalence of a particular vc type is significantly dependent on the population of origin – Kast population is mostly abundant with EU-2 and other vc types, while Ozalj contained more of the vc-type EU-1 (Supplementary Table 1), which might have contributed to the observed specificities of the stress level indicators.

The absolute values of laccase activity in samples of *C. parasitica* naturally infected with CHV1 measured in this study fell exclusively within the lower end of the range of values observed by Nuskern et al. (2021) in samples which have been infected with CHV1 by laboratory transmissions (Supplementary Table 2). It was possible to directly compare these two studies as the experimental setup was the same. This is important as the laccase activity is dependent of the experimental setup, e.g., the cultivation medium used, duration of cultivation and equipment used in experiment (Nuskern et al., 2021). This also applies to the studies that measured laccase mRNA accumulation – the level of accumulation was proven to be influenced by the culture medium, the age of the culture and exposure to the light (Choi et al., 1992). Importantly, *lac1* expression was assessed almost exclusively on prototypic CHV1 strain EP713 and its isogenic virus-free fungal isolate EP155 (Rigling and Van Alfen, 1991; Choi et al., 1992; Larson et al., 1992; Rigling and van Alfen, 1993; Kazmierczak et al., 1996; Allen et al., 2003). This makes our study unique: to the best of our knowledge *lac1* transcription levels have not been estimated in CHV 1-infected *C. parasitica* strains derived from the wild, but rather only from the aforementioned prototypic strains.

Our research indicated that the factor determining laccase physiology is intrinsic to the fungus itself, i.e., not related to CHV1 intra-host diversity nor the fungal population from which the samples have been obtained. Accordingly, we did demonstrate the dependence of both *lac1* expression and laccase activity on the vegetative compatibility (vc) type of the fungus. As other filamentous Ascomycetes (Saupe, 2000), *C. parasitica* also possesses a heterokaryonic or vegetative compatibility (vc) system, that is a self–non-self-recognition system controlling the stability of anastomoses and heterokaryon formation. Vegetative compatibility is controlled by six known diallelic *vic* loci, defining 64 different vc types, also known as EU-types (Cortesi and Milgroom, 1998; Robin et al., 2000; Cortesi et al., 2001). It seems that laccase expression is regulated at a different pace in vc types EU-1 and EU-2: the expression levels of *lac1* in EU-1 were lower, compared to the expression of *lac1* in EU-2. The enzymatic activity of laccase was different as well between vc-types EU-1 and EU-2, but it was higher in EU-1 and lower in EU-2. Similarly, the lower lipid oxidation and higher concentration of non-protein thiols found in EU-2 compared to EU-1 suggest that the vc type (i.e., *vic* genotype) of the fungus might be an important physiological determinant. Since proteins with diverse domains are encoded by the *vic* loci (Choi et al., 2012; Dawe and Nuss, 2013; Zhang et al., 2014; Paoletti, 2016) they might be involved in several cellular processes beyond cell death/barrage formation, leading to the phenomenon observed. Specifically, the vc types EU-1 and EU-2 differ at locus *vic2,* which is responsible for the strongest incompatibility reaction (Cortesi et al., 2001), and that encodes for the patatin-like lipase and sec9 plasma membrane protein (Choi et al., 2012). In other fungal species, it was proposed that these protein analogs are involved in allorecognition, i.e., sec9 protein might bind to patatin-like phospholipase 1 and activate downstream metabolic response (Heller et al., 2018) which results in programmed cell death. Our results add to the slowly growing body of evidence (Leigh et al., 2021), that suggests that the aforementioned genes/proteins might be involved in another role beyond self/non-self recognition.

## 5. Conclusion

In our study, we observed lower enzymatic activities of SOD and GST and higher content of MDA and total non-protein thiols in model CHV1-infected *C. parasitica* isolates than in CHV1-infected isolates freshly obtained from wild populations. This indicates higher oxidative stress in the former, which can have serious implications if generalized inferences were to be drawn based solely on such model strains.

Furthermore, we observed significant differences between stress levels of the wild populations, underlying particular genetic distinctiveness of natural populations. While CHV1 intra-host diversity had no discernible effect, a particular vegetative incompatibility locus of the fungus host was shown to influence both *lac1* expression and laccase enzyme activity.

## Data availability statement

The original contributions presented in the study are included in the article/Supplementary material, further inquiries can be directed to the corresponding author.

## Author contributions

MP and LN: physiology experiments, data analysis, and writing. KP: fungal culture preparation and physiology experiments. RV, ZK, and LK: expression analyses and writing. MĆ-P, DL, IP, and MI: writing. DR and MJ: experimental design, writing, and funding. All authors contributed to the article and approved the submitted version.

## Funding

This research was funded by the Swiss Enlargement Contribution in the framework of the Croatian-Swiss Research Programme (project number IZHRZ0_180651), Croatian Science Foundation (grant number IP-2018-01-1295), and University of Zagreb financial support.

## Conflict of interest

The authors declare that the research was conducted in the absence of any commercial or financial relationships that could be construed as a potential conflict of interest.

## Publisher’s note

All claims expressed in this article are solely those of the authors and do not necessarily represent those of their affiliated organizations, or those of the publisher, the editors and the reviewers. Any product that may be evaluated in this article, or claim that may be made by its manufacturer, is not guaranteed or endorsed by the publisher.
