# Supplementary material for: Physiological variations in hypovirus-infected wild and model long-term laboratory strains of Cryphonectria parasitica
Source: Front Microbiol. 2023 Jun 22;14:1192996. doi: 10.3389/fmicb.2023.1192996 (PMC10324583; doi:10.3389/fmicb.2023.1192996)
Supplement: Supplementary file 1 [file Data_Sheet_1.docx]

Supplementary Material

Physiological variations in hypovirus-infected wild and model long-term laboratory strains of *Cryphonectria parasitica*

Maja Popović^†^, Lucija Nuskern^†^, Karla Peranić, Rosemary Vuković, Zorana Katanić, Ljiljana Krstin, Mirna Ćurković-Perica, Deborah Marie Leigh, Igor Poljak, Marilena Idžojtić, Daniel Rigling, Marin Ježić^*^

*** Correspondence:** Marin Ježić: marin.jezic@biol.pmf.hr

# Supplementary Figures and Tables

## Supplementary Figures


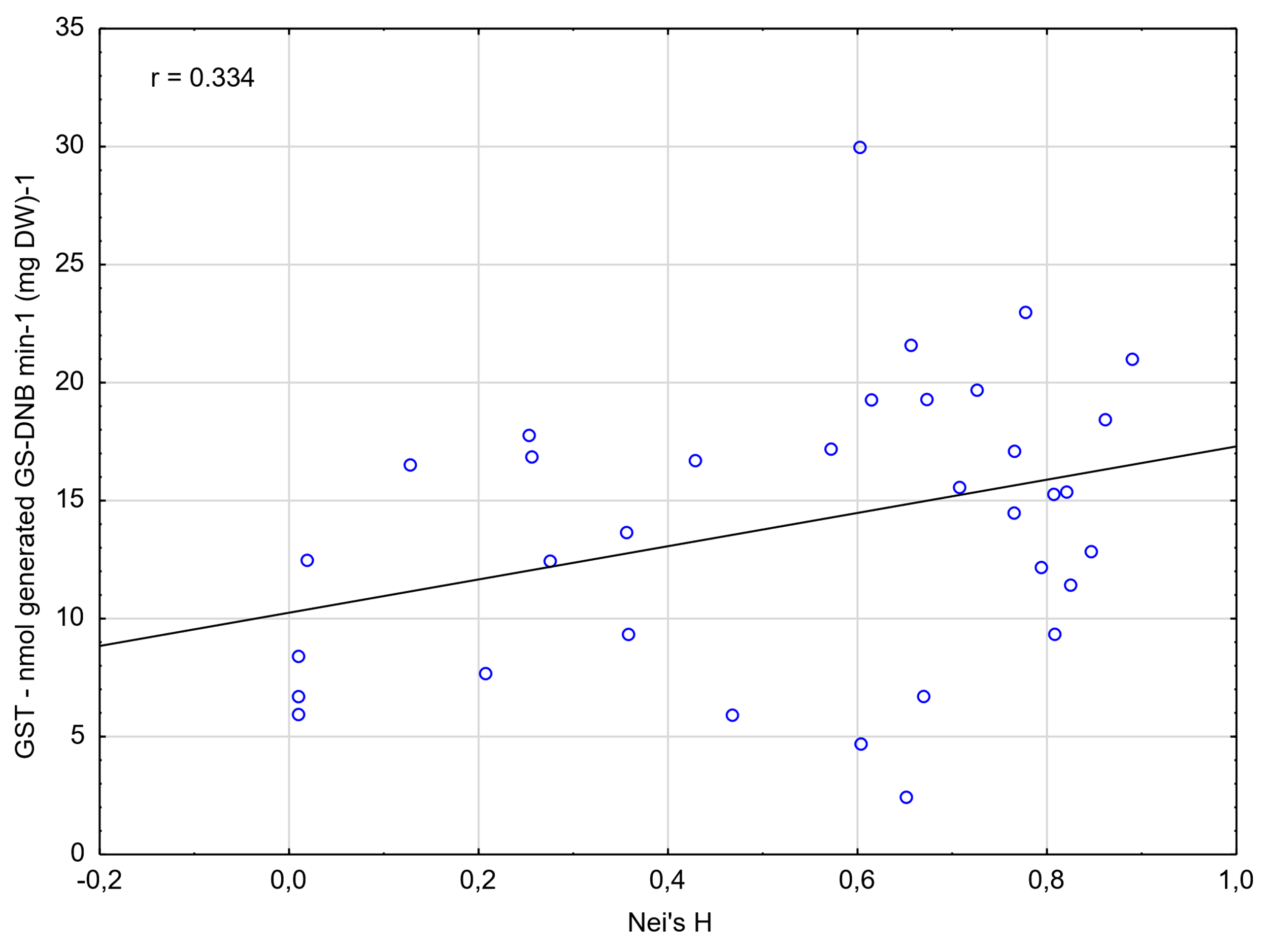


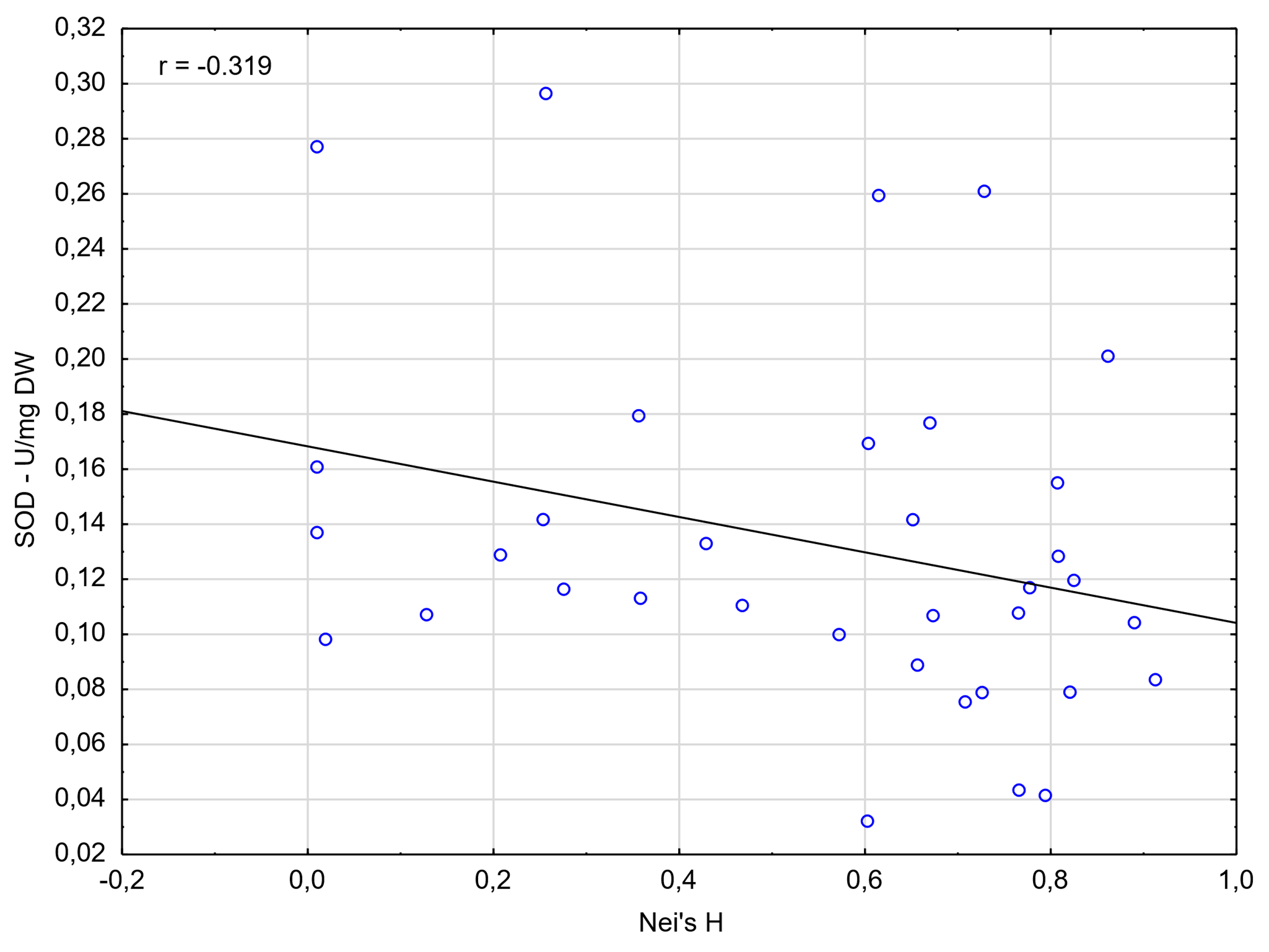


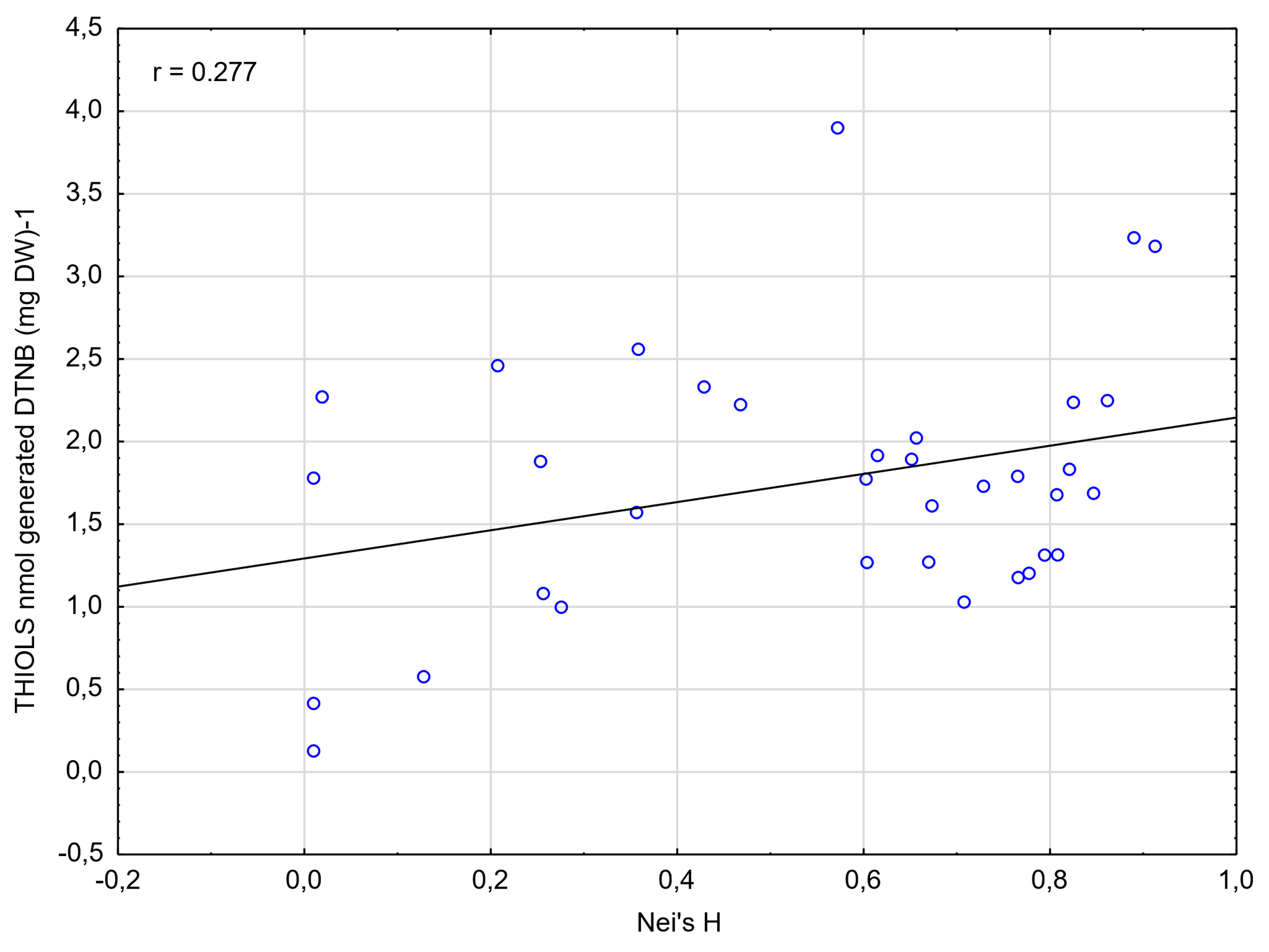


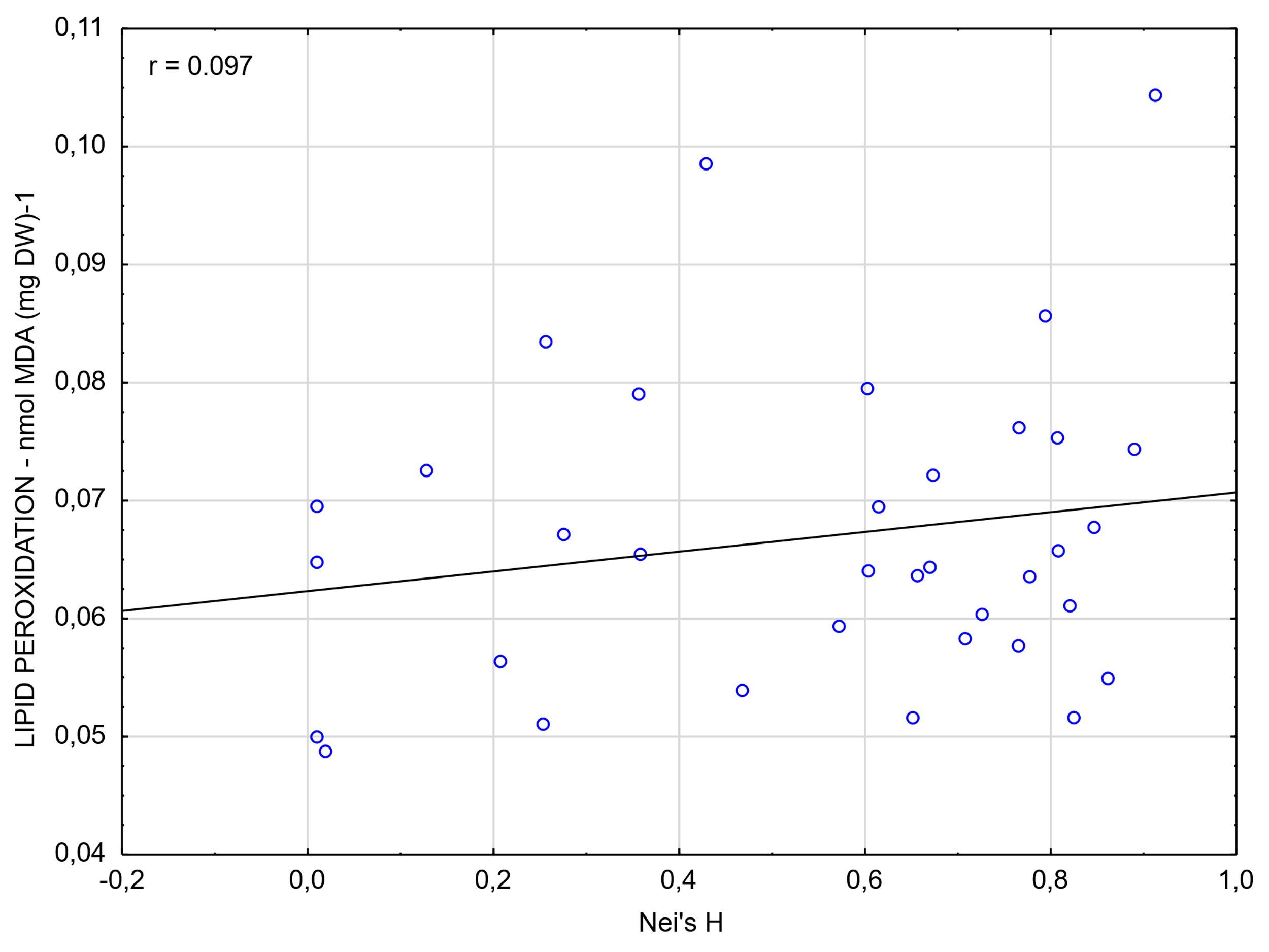


**Supplementary Figure 1.** Pearson's correlation coefficient between Nei’s H and oxidative stress parameters; * asterisks denote statistically significant correlations at p < 0.05.
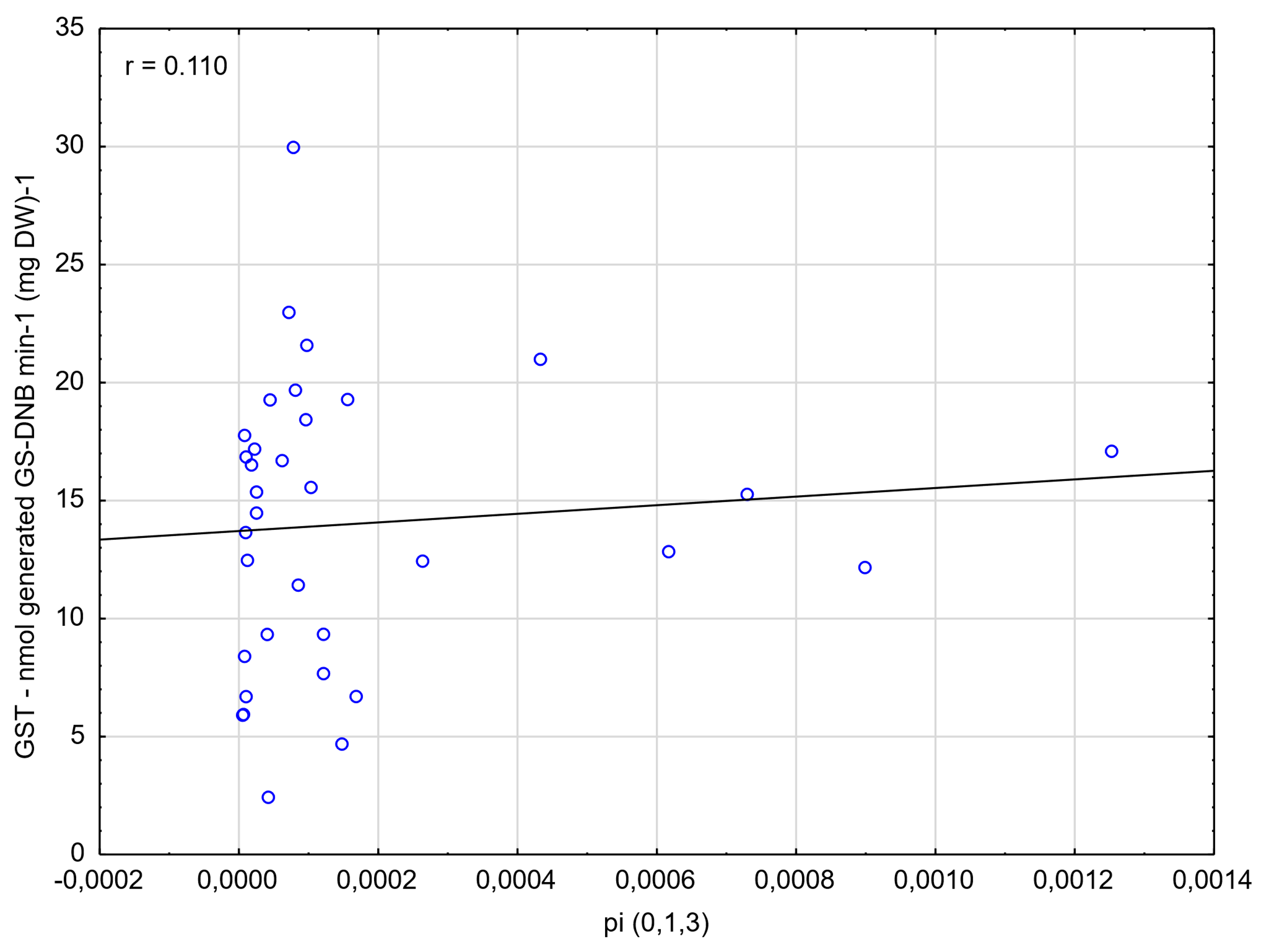


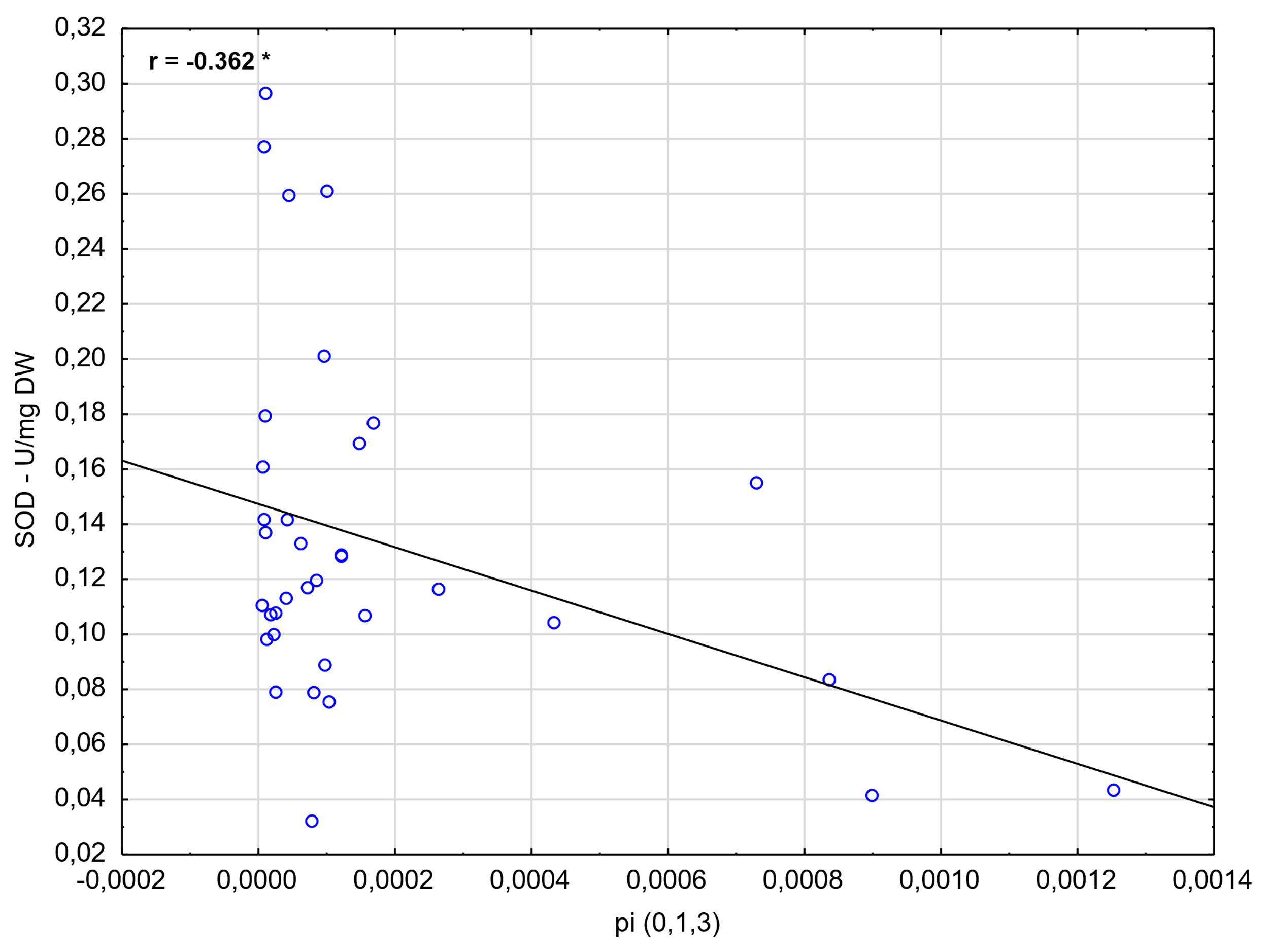


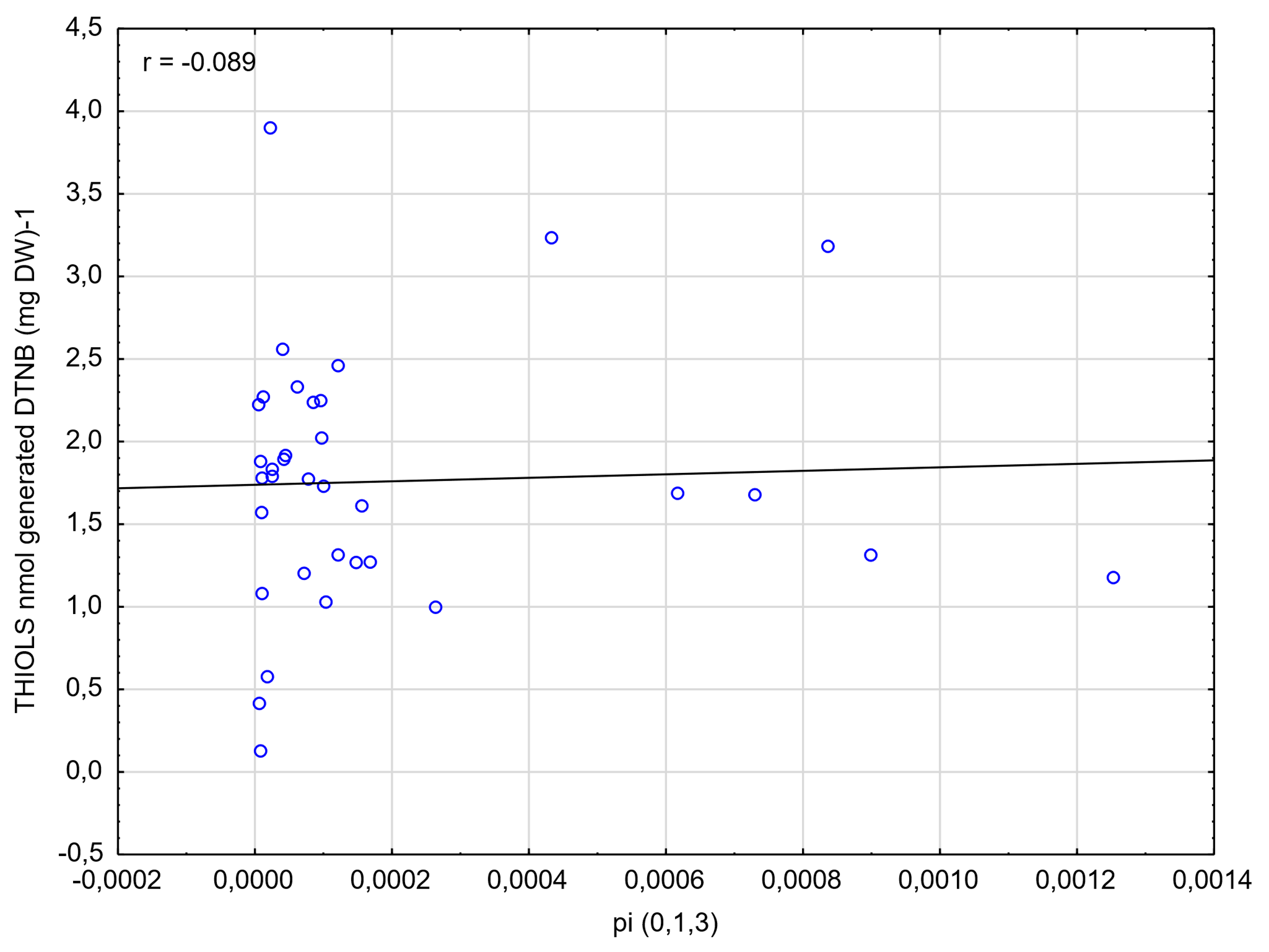


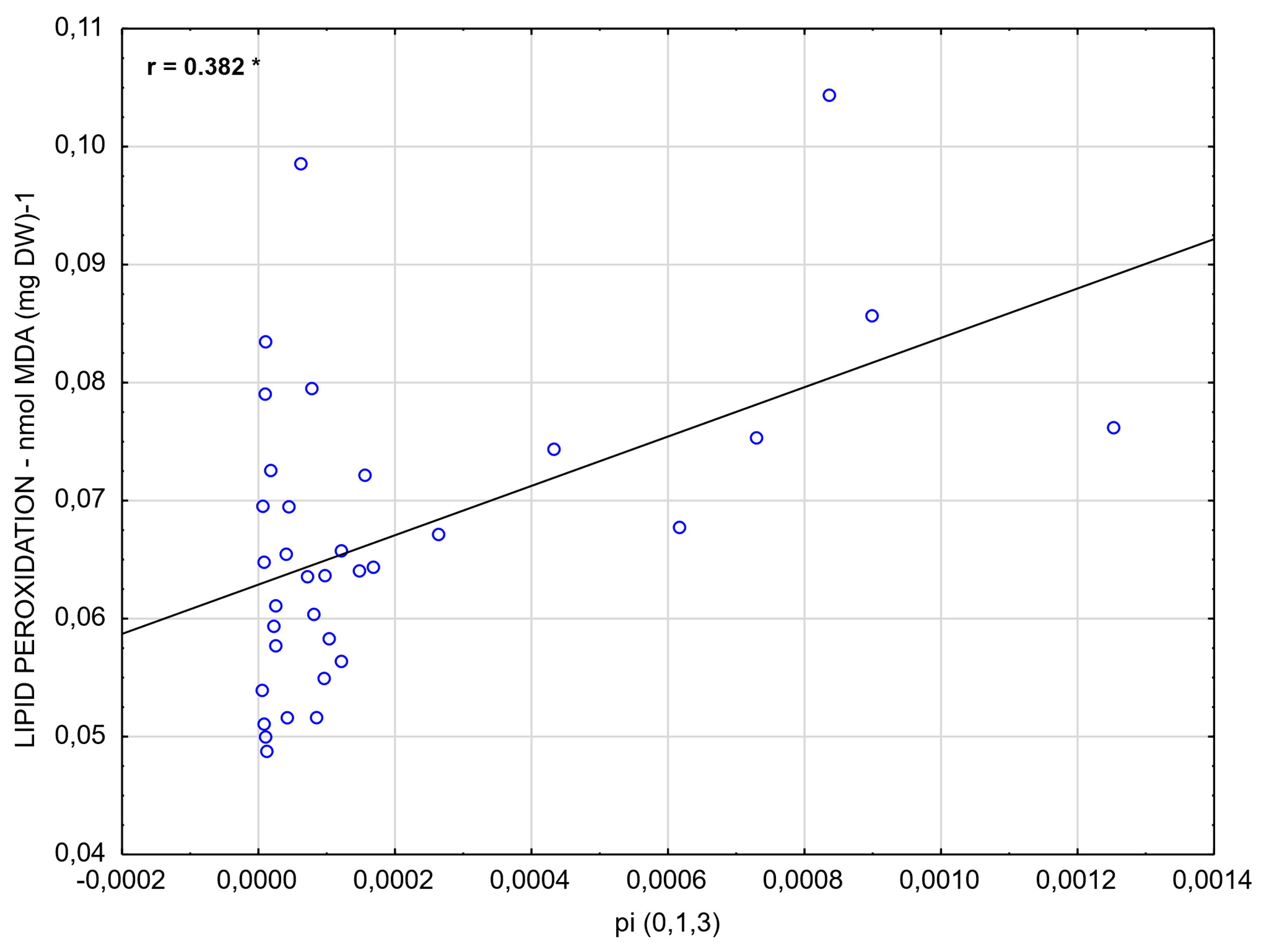


**Supplementary Figure 2.** Pearson's correlation coefficient between π and oxidative stress parameters; * asterisks denote statistically significant correlations at p < 0.05.

## Supplementary tables

Supplementary table 1. Oxidative stress parameters measurement for all analyzed samples

Supplementary table 2. Laccase activity and lac1 relative expression for all analyzed samples
